# Temporal cross‐correlation between influenza‐like illnesses and invasive pneumococcal disease in The Netherlands

**DOI:** 10.1111/irv.12442

**Published:** 2017-01-03

**Authors:** Wilke Hendriks, Hendriek Boshuizen, Arnold Dekkers, Mirjam Knol, Ge A. Donker, Arie van der Ende, Hester Korthals Altes

**Affiliations:** ^1^ Centre for Infectious Disease ControlNational Institute for Public Health and the EnvironmentBilthovenThe Netherlands; ^2^ Department for StatisticsInformatics and ModelingNational Institute for Public Health and the EnvironmentBilthovenThe Netherlands; ^3^ NIVEL Primary Care DatabaseSentinel PracticesUtrechtThe Netherlands; ^4^ Department of Medical MicrobiologyAcademic Medical CenterCenter for Infection and Immunity AmsterdamAmsterdamThe Netherlands; ^5^ Netherlands Reference Laboratory for Bacterial MeningitisAmsterdamThe Netherlands

**Keywords:** cross‐correlation, filtering, influenza, pneumococci, pre‐whitening, respiratory tract infections, SARIMA

## Abstract

**Background:**

While the burden of community‐acquired pneumonia and invasive pneumococcal disease (IPD) is still considerable, there is little insight in the factors contributing to disease. Previous research on the lagged relationship between respiratory viruses and pneumococcal disease incidence is inconclusive, and studies correcting for temporal autocorrelation are lacking.

**Objectives:**

To investigate the temporal relation between influenza‐like illnesses (ILI) and IPD, correcting for temporal autocorrelation.

**Methods:**

Weekly counts of ILI were obtained from the Sentinel Practices of NIVEL Primary Care Database. IPD data were collected from the Dutch laboratory‐based surveillance system for bacterial meningitis from 2004 to 2014. We analysed the correlation between time series, pre‐whitening the dependent time series with the best‐fit seasonal autoregressive integrated moving average (SARIMA) model to the independent time series. We performed cross‐correlations between ILI and IPD incidences, and the (pre‐whitened) residuals, in the overall population and in the elderly.

**Results:**

We found significant cross‐correlations between ILI and IPD incidences peaking at lags ‐3 overall and at 1 week in the 65+ population. However, after pre‐whitening, no cross‐correlations were apparent in either population group.

**Conclusion:**

Our study suggests that ILI occurrence does not seem to be the major driver of IPD incidence in The Netherlands.

## 1 Introduction

Invasive pneumococcal disease (IPD) is the infectious disease with the highest average disease burden in the Netherlands.^1^ It shows a seasonal pattern peaking in winter,^2^, ^3^ but little is known about the driver of this pattern.^4^ Contributing factors proposed include climate variables,^5^ air pollution^6^ and cocirculating respiratory viruses. In particular, influenza and respiratory syncytial virus (RSV) 7, 8 are suspected to predispose to pneumococcal infection.^9^, ^10^

Several animal studies document a significant association between pneumococcal infections following an influenza infection, enhancing pneumococcal disease severity^11^ and susceptibility.^12^, ^13^ Histopathological studies in deceased and severe human influenza cases during a pandemic outbreak^14^, ^15^, ^16^ attribute a substantial fraction of influenza deaths to secondary pneumococcal infection. The strength of the interaction between influenza or influenza‐like illness (ILI) and IPD at population level, however, varies across studies from non‐existent to significant.^17^, ^18^, ^19^, ^20^, ^21^, ^22^, ^23^, ^24^, ^25^, ^26^, ^27^ Studies looking at specific respiratory viruses also showed inconsistent results.^5^, ^28^, ^29^, ^30^ In addition, most studies did not control for seasonality of the pathogens or used a sine/cosine function to adjust for it.^27^ However, this function might not give the best fit to seasonal trends in infectious diseases: in influenza, for example, the seasonal pattern is a yearly spike, rather than a symmetrical (co‐)sine curve around the yearly mean. The common seasonal trend of ILI and IPD makes it difficult to interpret the correlations, as a similar seasonal pattern could reflect a common seasonal driver.

Therefore, we wish to explore the correlation in time between ILI and IPD in the Netherlands at population level, using time‐series analyses correcting for temporal autocorrelation within time series. A method termed pre‐whitening adjusts for autocorrelation within time series.^31^ It involves using the best‐fit autoregressive model to the independent time series, to filter the dependent time series. This then allows to correlate the residuals of the best‐fit model to the independent time series with the residuals of the dependent time series (IPD) filtered with this best‐fit model. This has been used successfully in areas such as ecology, for example, to quantify the lag between fishing pressure and fish population size.^32^ We compared the plain correlation function between the ILI and IPD time series, without adjusting for (seasonal) autocorrelation, with the correlation function after pre‐whitening. Because temporal patterns in IPD might differ between age groups, we also investigated this temporal correlation in the subpopulation of people aged 65 and older.

## 2 Methods

### 2.1 Study setting and population

We conducted an ecological study, comparing weekly incidence rates of ILI and IPD in the Dutch population from week 1 in 2004 to week 25 in 2014. We then focused on different age categories, as factors driving pneumococcal disease development and vaccination effects might differ across age groups. When plotting IPD incidence data by age category (Fig. S1), we find that only the subgroup of people aged 65 year or older (“elderly”) has high enough incidence to allow for a subgroup analysis.

### 2.2 ILI and IPD data sources

**Figure 1:**
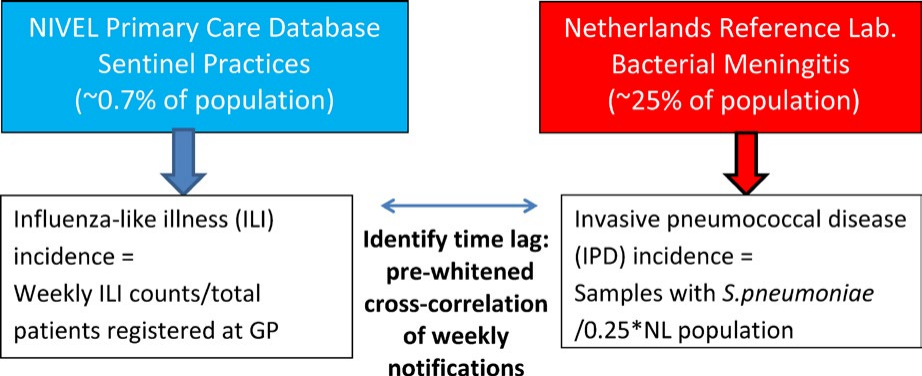
Schematic of databases used for weekly ILI and IPD incidence calculations

In the Netherlands, the weekly number of ILI cases was obtained from the Sentinel Practices of NIVEL Primary Care Database. This sentinel network of general practitioners (GPs) covers 0.7% of the total Dutch population (Figure 1). It is representative in age, gender, regional distribution and population density.^33^ A reported ILI case was defined as a person who contacted the GP and was diagnosed with ILI according to the criteria of Pel: an acute start (maximum prodromal stage of 3‐4 days) of at least one of the following symptoms: cough, nasal catarrh, sore throat, frontal headache, retrosternal pain or myalgia, accompanied by a rectal temperature of at least 38°C.^34^ The weekly incidence of ILI cases was calculated by dividing the reported ILI patients by the total number of patients registered by the participating GPs.

IPD data were obtained from the laboratory‐based surveillance system Netherlands Reference Laboratory for Bacterial Meningitis (NRLBM). It receives isolates from blood and cerebrospinal fluid (CSF) of patients with IPD from nine evenly distributed sentinel laboratories (Figure 1) covering 25% of the population. IPD was defined as a case in which *S. Pneumoniae* was isolated from blood or CSF, so it was not limited to meningitis.^35^ The date of blood sampling at the hospital was missing in 0.7% of the records. We used date of sample reception at the laboratory as proxy for IPD notification date. This does result in a mean delay of 7 days in IPD case reporting, as there is approximately 7 days between sampling and testing at the local hospital, and reception for serotyping at the Reference Laboratory for Bacterial Meningitis (data not shown).

The population data of the Netherlands were obtained from statistics Netherlands.^36^

### 2.3 Vaccination

The influenza vaccine indication was revised in 2008, lowering the recommended age to start seasonal influenza vaccination from 65 to 60 years.^37^ Influenza vaccination coverage of the total population during 2004‐2013 was on average 20%. In the elderly (≥60 years), coverage declined after 2008 from 82.5% to 72.2% in 2013.^38^, ^39^ We do not expect these changes in vaccination policy and coverage to substantially affect ILI notifications in the elderly population. We have not taken into consideration influenza vaccination, mainly because we do not expect it to affect the relation between ILI and IPD.

Pneumococcal conjugate vaccination against carriage of and infection with seven subtypes of *S. Pneumoniae* (PCV7) was introduced in the National immunization program (NIP) for all infants born from 1 April 2006 onwards in the Netherlands. From 1 March 2011 onwards, all infants received a vaccination against ten subtypes instead of seven (PCV10). The NIP vaccination uptake each year is on average 94.8%.^40^ The 23‐valent pneumococcal polysaccharide vaccine is not routinely recommended for the older population, and its uptake therefore is negligible.^41^

To assess whether PCV7 vaccination was a possible confounder influencing IPD notifications over the study period, we tested whether IPD incidence changed after vaccine introduction. An effect of PCV7 vaccine introduction occurs at different moments in time, depending on the age category considered, due to the cohort effect. We therefore have to split the IPD notifications according to age (Fig. S1); only in the elderly population do we have high enough IPD incidence to assess a vaccine effect. We examined whether there was a possible delayed effect of PCV7 vaccination, introduced in children in 2006, on IPD incidence in the elderly population, by fitting a generalized linear mixed model to the IPD counts in the elderly population corrected for population size (GLIMMIX procedure, SAS). We assumed Poisson distributed counts, adjusted for population size by including the logarithm of the yearly (over 65 years old) population as offset. Autocorrelation in time was included in the model, and the seasonality was described through a half‐sine function. See Supplement section A for details.

### 2.4 Data transformation and time‐series correlation

**Figure 2:**
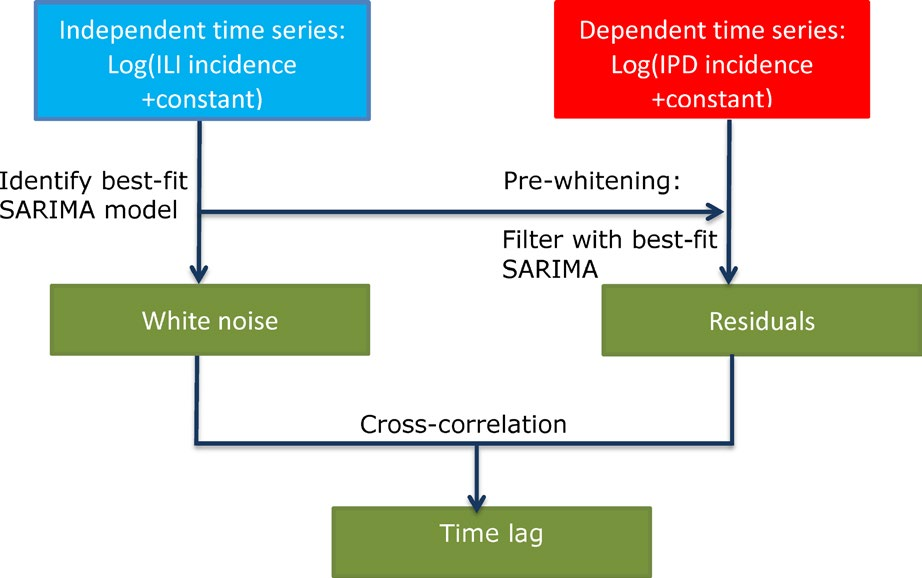
Schematic of pre‐whitening approach for cross‐correlation analysis. Both time series are transformed to stabilize the variance; the best‐fit SARIMA model to the independent variable is then used to filter the dependent variable. The white noise of the best‐fit SARIMA and the residuals of the filtered dependent time series are cross‐correlated. Lags with a significant correlation will indicate the time lag between the independent and dependent time series

Weekly ILI and IPD incidences were cross‐correlated using the cross‐correlation function (CCF). Then, the CCF was used after applying the pre‐whitening method,^42^ which corrects for autocorrelation within time series: this is achieved by fitting a seasonal autoregressive integrative moving average (SARIMA) model^43^ to the independent time series—ILI—and filtering the dependent time series—IPD—with the best‐fit model of the independent time series (Figure 2). This removes the autocorrelation present in the independent series from the dependent series. Any remaining correlation between the series then can no longer be due to a common autocorrelation structure, including the seasonality, as the seasonal pattern in SARIMA is modelled as an autocorrelation between measurements exactly 1 year apart. The method for SARIMA fitting and for pre‐whitening is explained in more detail in Supplement, section B and C, respectively. Finally, a CCF was plotted between the residuals of the best‐fit SARIMA model to the ILI data, and the filtered IPD data (Figure 2). For the analysis in the elderly, the same procedures were used over the period of week 38 in 2004 to week 25 in 2014: the first weeks in 2004 were excluded because in many weeks, no ILI cases were found.

These statistical analyses were performed with RStudio (version 0.99.441), and software R (version 3.2.0). Packages used were: foreign, MASS, plyr, ISOweek, astsa and forecast.

## 3 Results

**Figure 3:**
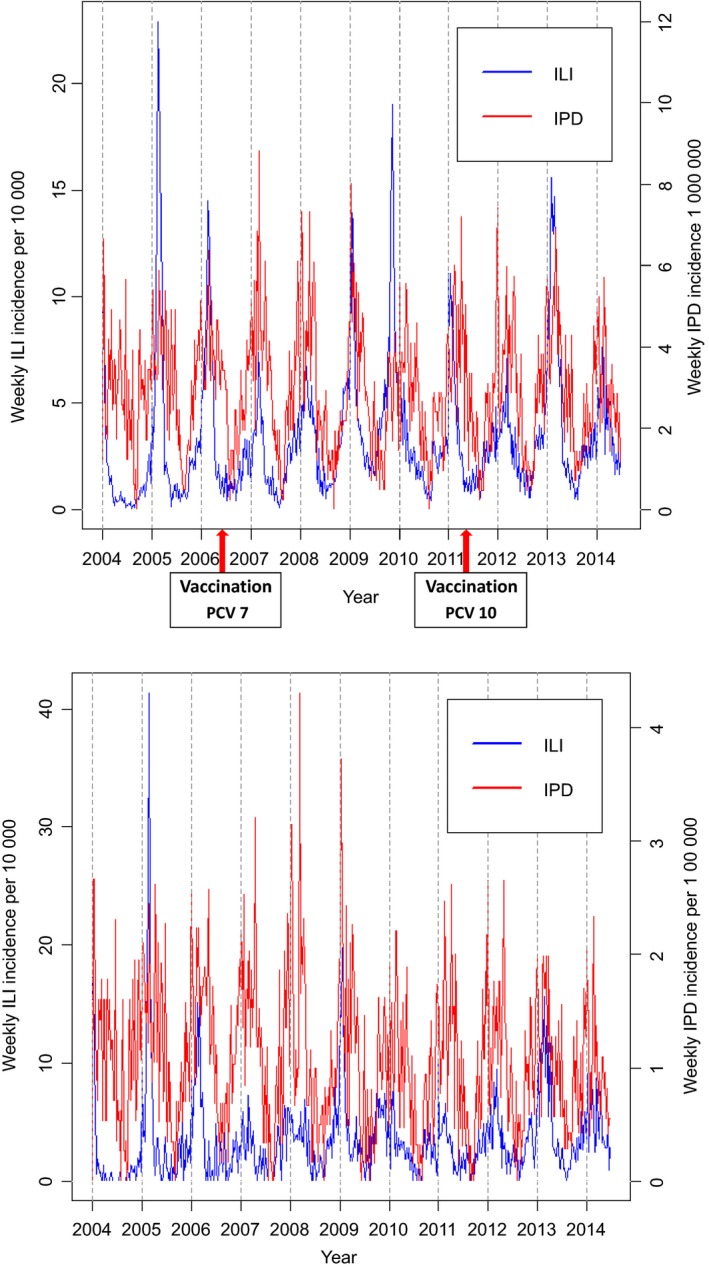
Weekly incidence of influenza‐like illnesses and invasive pneumococcal disease recorded in the Dutch population 2004‐2014 (A) across all age groups (B) aged 65 years and over

Between 2004 week 1 and 2014 week 25, a total of 20,601 ILI and 6,572 IPD cases were recorded through the NIVEL network and bacterial meningitis database (with coverage 0.7% and 25%, respectively). In the subpopulation aged 65 years and over, 3,202 ILI and 3,399 IPD cases were recorded between week 38 in 2004 and week 25 in 2014. ILI and IPD incidences show a shared seasonal periodicity, peaking during the winter weeks (Figure 3A). Note that incidences for ILI and IPD are given per 10 000 and 1 000 000 inhabitants, respectively. An early ILI peak appears at the end of 2009, the year the influenza AH1N1 pandemic occurred. The ILI incidence peaks varied in time of onset, height and duration between years, while the IPD incidence shows a seasonal pattern that is more constant. In the population aged ≥65 years, the same patterns are seen (Figure 3B). Typically, the height of ILI peaks in the older group differs from the peak height in the overall population in the same year.

The generalized linear mixed model fit shows that the variable catching the effect of pneumococcal vaccine (PCV7) introduction is not significant, nor the interaction between time in weeks and vaccine introduction (“vaccination” and “time*vaccination” in Table 1). Thus, the analysis of the effect of vaccination on IPD incidence in the population of people aged 65 years or older does not suggest a change in notifications, 2 years after introduction of infant IPD vaccination.

**Table 1:** Fixed effect variables of fitted generalized linear mixed model to the weekly IPD notifications in the population 65 years and over (output of GLIMMIX, SAS procedure); delayed effect of vaccination

Solutions for fixed effects					
Effect	Estimate	Standard Error	*df*	t Value	Pr>|t|
Intercept	−13.7079	0.1547	0	−88.61	.
Time	−0.0004	0.0004	539	−1.18	0.2374
Vaccination	−0.0207	0.1613	539	−0.13	0.8981
Seasoncom	1.3567	0.0944	539	14.37	<.0001
Time*Vaccination	0.0008	0.000650	539	1.17	0.2433

**Figure 4:**
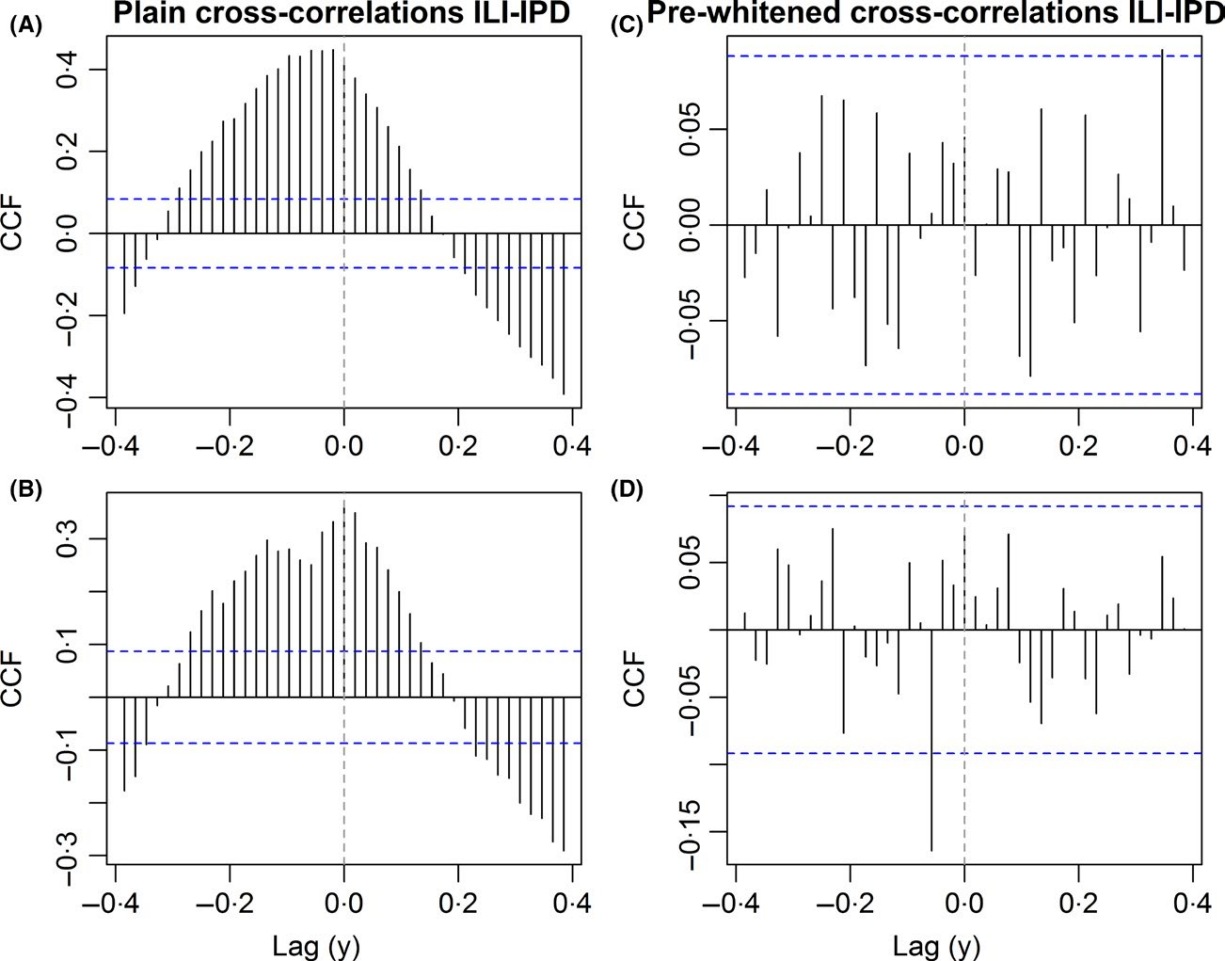
Cross‐correlation functions between weekly ILI and IPD incidences (A) in the whole population (B) for elderly (65+) population; pre‐whitened cross‐correlation functions for same respective groups in (C)—whole population and (D)—elderly, on log‐transformed data. The 95% confidence intervals around 0 are indicated by the dashed blue lines

The plain cross‐correlations between ILI and IPD incidences in the whole population are highest at lags between −3 and −1, reaching 0.45 (Figure 4A): ILI incidences show greatest correlation with IPD incidences 1‐3 weeks later. Likewise, the cross‐correlation in the older age group is highest at lags between ‐2 to 1 week (Figure 4B).

To accomplish pre‐whitening, the best‐fit SARIMA model was selected by examining the ACF and PACF plots of the independent (ILI) time series. Section B in the supplement details the fitting procedure of the SARIMA model. Section D in the supplement shows the residuals of the best‐fit SARIMA to the ILI time series and the residuals of the filtered IPD time series, as these are the basis of the pre‐whitened CCF. The cross‐correlations found between the incidences (Figure 4A) are absent after pre‐whitening (Figure 4C). The correlations fluctuate randomly around zero, and no significant lags are apparent. The pre‐whitened cross‐correlations in the 65+ population show no significance, except the correlation at lag ‐3 weeks (Figure 4D). This value has the opposite sign of the correlation that is logically expected from IPD being a complication of ILI, so we may attribute this to multiple testing error (Figure 4D).

## 4 Discussion

The literature on the lagged relationship between incidence of respiratory viruses and pneumococcal disease incidence is inconclusive, and studies adjusting for the autocorrelation within time series are lacking. Using data from two surveillance systems within the Dutch population over a 10‐year period, we confirmed seasonality in both ILI and IPD incidences. When correcting for temporal autocorrelation, we found no correlation between the residual time series of ILI and IPD in the overall population, nor in the population over 65 years of age. While the ILI peaks varied strongly from season to season, IPD incidence showed a more stable seasonal pattern, as observed elsewhere.^19^ An important event during the study period is the implementation of infant vaccination against IPD (PCV7) through the Dutch national immunization program in 2006. In the United States, a decline in IPD cases was detected post‐vaccination in the non‐vaccinated elderly, referred to as indirect immunity.^44^ A study comparing cumulative IPD incidences over the pre‐vaccination years 2004‐2006 and late post‐vaccination period 2008‐2010 suggested a decline in IPD incidence in infants (age<2 years) and elderly (>65 years).^45^ If real, such an effect of vaccination would obscure possible cross‐correlations between ILI and IPD incidences. However, we failed to reproduce this effect, when using a regression model allowing for an autocorrelation term on the entire 2004‐2014 time series, testing for a break in trend in 2008. It seems probable that an underlying time trend gives the effect found when comparing the 2004‐2006 with the 2008‐2010 incidences.^45^ It is, therefore, unlikely that vaccination affected a possible cross‐correlation between ILI and IPD incidences.

Without correcting for seasonal patterns and other temporal autocorrelations, significant cross‐correlations were found between ILI and IPD, with highest correlations from lag ‐3 to ‐1 week. The subpopulation of people aged 65 years and older, the age category with most IPD cases, also shows significant cross‐correlations between ILI and IPD highest between lag ‐2 to 1 week. After pre‐whitening, the cross‐correlations between the ILI and IPD residuals were not significant anymore. The same was found in the population over 65 years of age.

The one‐week lag observed between ILI and IPD through plain CCF corresponds to the lag observed at individual level.^11^, ^46^, ^47^ Our results are in line with those of Kuster et al.^19^ This Canadian study finds that influenza notifications Granger‐cause IPD, consistent with our observed lagged relation by plain cross‐correlation analysis, but yearly phase and amplitude terms of the fitted sine waves to the two time series were not correlated^19^—paralleling our result that (pre‐whitened) residuals were not correlated. Actually, most studies not correcting for autocorrelation report a temporal association between either influenza and/or RSV with IPD incidence.^5^, ^18^, ^20^, ^25^, ^28^, ^29^ Strikingly, the study in children up to 16 years of age by Toschke et al., taking into account temporal autocorrelation, showed no association between influenza outbreaks and invasive pneumococcal infection.^27^ Similarly, the association between influenza and *S. Pneumoniae* disappears when taking into account seasonal patterns.^48^

Shrestha et al. fitted a between‐host transmission model of influenza and pneumococcal infection to surveillance data, to identify the most likely mechanism of interaction.^49^ They argue that the roughly one‐week lag observed between influenza and pneumococcal pneumonia is most likely attributable to influenza‐enhanced disease susceptibility.^49^ Interestingly, the interaction term could only be inferred from simulated influenza and IPD time series, when the yearly variability in seasonal influenza peak height was considerable, that is a more than fourfold difference in peak height from one season to the next.^49^ In our study, the seasonal influenza peak in the population as a whole does not show the “required” variability for a detectable effect on IPD incidence, but for the elderly population, it does (*eg* 2005 vs 2007 epidemic). Yet, we do not pick up a signal through pre‐whitened cross‐correlation. It would be interesting to see what level of simulated interaction in a mechanistic model would lead to a measurable effect (*ie* a significant lag) through a pre‐whitened CCF study between the two interacting simulated time series.

The ILI peak at the end of 2009, corresponding to the influenza A‐H1N1 pandemic, is notably earlier than the regular ILI peaks. During this pandemic with early onset, IPD incidence remained low and increased later, following its own seasonal pattern. It is known that this A‐H1N1 pandemic affected an unusual population subset, with more younger and fewer elderly cases.^50^ Consistent with Kelly et al.*,* 50 the ILI peak corresponding to the pandemic of 2009 is relatively smaller in the subgroup of people aged 65 and over. To overcome the problem that different age groups might show different seasonal variation, analyses should be carried out in age‐stratified subpopulations. In this study, only the age category of 65 years and older had high enough weekly IPD incidence for analysis. Still, the cross‐correlation between the pre‐whitened ILI and IPD incidence time series in the elderly was not significant.

In fact, associations reported in the literature were often not only age‐dependent, but also virus‐specific. RSV and IPD are associated in infants/children,^5^, ^17^, ^26^, ^29^, ^30^ and influenza and IPD in the elderly.^26^, ^28^, ^30^ The strongest associations between respiratory viruses and pneumococcal disease occur among the older age groups.^18^, ^20^, ^22^, ^26^ We could not analyse RSV nor influenza incidence: routine virological testing in the Netherlands, carried out only on sampled ILI patients, gives insufficient power for a time‐series analysis. Possibly, we miss a temporal correlation between ILI and IPD in the elderly, as there might be more noise in ILI notifications, due to the large proportion of non‐influenza ILI, than in influenza notifications. The correspondence between ILI and influenza incidence is at its best at ILI peak, with a positive predictive value around 50%^49^; as one moves away in time from the peak, the proportion of influenza‐related ILI drops substantially.^51^ This would imply that the influenza peak is narrower than the ILI peak. As IPD notifications already tend to show a broader yearly peak than ILI, we would not expect the association between influenza and IPD to become stronger. RSV likely affects proportionately more children (95%, see 20), so we do not expect a strong association between RSV and IPD in the elderly. Interestingly, Watson et al. found only an interaction between combined influenza and RSV notifications and IPD in the whole population, while the association between RSV notifications only and IPD in children was significant.^30^

Lastly, IPD is associated with different clinical syndromes (meningitis, invasive pneumonia, etc.), each with a different genesis. It is possible that infection with influenza predisposes to one clinical syndrome rather than another: interaction through damage of the tissues in the respiratory tract would yield an association of ILI with pneumonia rather than with meningitis; interaction through a weakening of the immune response would yield an association between ILI and all syndromes. We cannot carry out the study on the subset of IPD cases associated with pneumonia, as we only have the clinical status up to 2012. However, the share of IPD due to pneumonia is about 75% in the period 2004‐2006 and 2008‐2012,^52^ and only 166 of the 6,572 IPD cases in our study have culture‐positive liquor, associated with meningitis. As most IPD in our data set is pneumonia‐related, we believe our result is unlikely to be flawed by a possibly reduced interaction with other clinical presentations of IPD other than pneumonia.

The pre‐whitening method removes the seasonal and other autocorrelative components of the time series compared, thereby also removing them from the cross‐correlation analysis. This way, the method tests whether a deviation from the fitted model (residuals) in one series correlates with the model deviation in the other series (residuals filtered series), one or more lags away. In this sense, pre‐whitening is a very strict test on cross‐correlation; however, it is a necessary transformation when testing for true association between autocorrelated time series 53. Using this method, we cannot rule out that the observed lagged association between ILI and IPD through plain CCF is due to a common seasonal driver, because the association is lost when carrying out a pre‐whitened CCF. In practice, this means that although we cannot rule out that ILI might predispose to IPD, ILI incidence is not a factor driving IPD incidence at population level. Probably, factors other than ILI incidence (more strongly) affect IPD incidence.

In summary, we found no correlation between the residual time series of ILI and IPD in the overall population, nor in the population over 65 years of age. Our study suggests that at population level, previous ILI occurrence does not seem to be the major driver of IPD incidence. An extension of our work would be to derive the pre‐whitened cross‐correlations between weekly notifications of influenza, RSV and IPD, in a population with a surveillance system for these pathogens, broken down by age. Also, the question of the detectability of possible interaction between respiratory viral infections and susceptibility to IPD through pre‐whitened CCF, as a follow‐up on Shrestha et al.'s work,^49^ might prove an interesting avenue of work.
