# Supplementary material for: Temporal cross‐correlation between influenza‐like illnesses and invasive pneumococcal disease in The Netherlands
Source: Influenza Other Respir Viruses. 2017 Jan 3;11(2):130–7. doi: 10.1111/irv.12442 (PMC5304567; doi:10.1111/irv.12442)
Supplement: Supplementary file 1 [file IRV-11-130-s001.docx]

**Supplement**

Figure S1: Weekly IPD case notifications in the Netherlands 2004-2014 across age categories (A) 0-4 years, (B) 5-14 years, (C) 15-49 years, (D) 50-64 years and (E) over 65 year-olds.

(B)


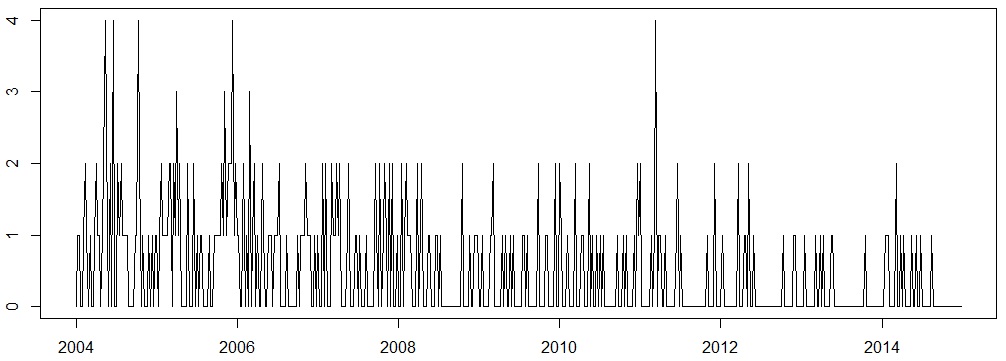


Weekly IPD notifications

(A)


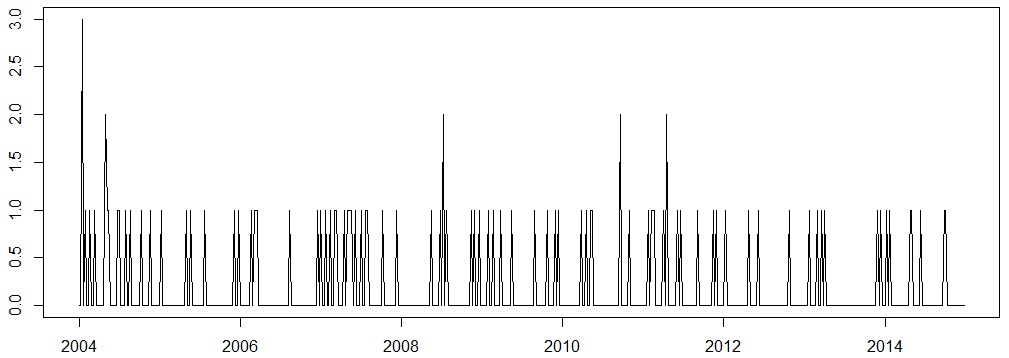


(C)


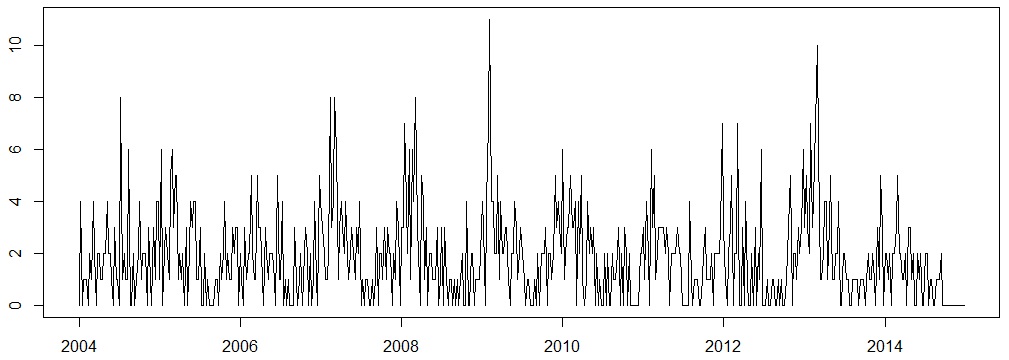


Time (years)


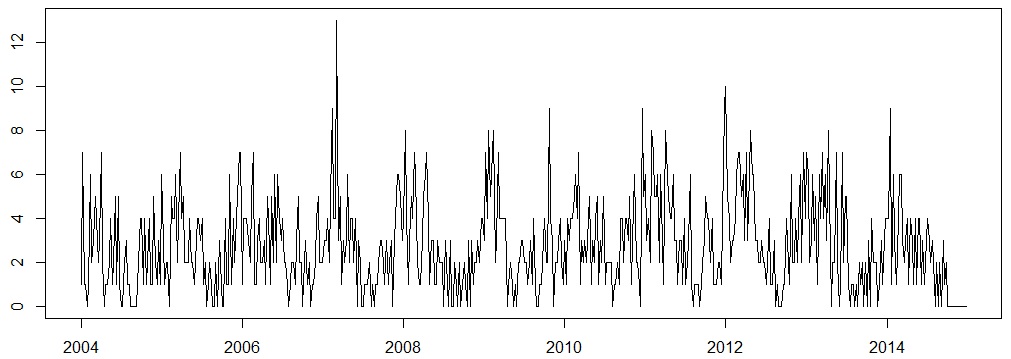

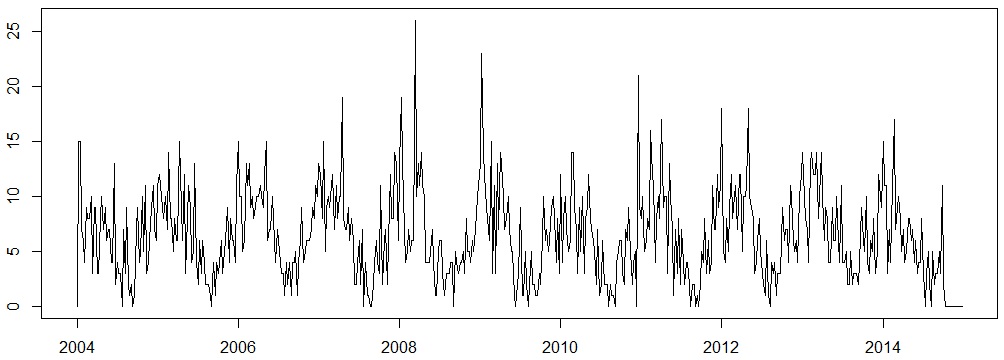


Weekly IPD notifications

(E)

(D)

Time (years)

**Section A: generalized linear mixed model to test for an effect of PCV7 vaccination on IPD incidence in elderly from mid 2008**

We set week number (w_S_) at 0 at the start of the IPD season, so when weekly IPD incidence is lowest, *i.e.* week 36.

So for the original weeknumber *w*:

If *w*≥36, *w_S_*=*w*-36;

If *w*<36, *w_S_*=*w*+16

The seasonal term S is expressed as

S(*w_S_*)= sinus[π(*w_S_*/52)]

So *S*(*w_S_*) is minimal at week *w*=36 and maximal at week *w*=10 (peak of the IPD season).

A variable was introduced referring to vaccine introduction (“*vaccination”*), which was set to 0 from week 1 in 2004 to week 20 in 2008, and 1 from week 21 in 2008 onwards.

Overall the equation for the generalized linear mixed model with an autoregressive order 1 (AR(1)) process is:

$log(E\left[ {IPDcount}_{time} \right])=\beta_{1}time + \beta_{2}vaccination + \beta_{3}S +\beta_{4} time*vaccination+log(populationsize)$

where *IPDcount* is the weekly number of IPD cases mong 65+, *time* is the time in weeks, *populationsize* is the size of the 65+ group, and the time series is obtained over *n* timepoints

and $\mathrm{var}\left[ IPDcount \right]=\gamma\mathbf{A}^{\frac{1}{2}}\mathbf{R} \mathbf{A}^{\frac{1}{2}}$

where $\boldsymbol{A}=\left( \begin{matrix} E[{IPDcount}_{1}] & \cdots& 0 \\ \vdots& \ddots& \vdots\\ 0 & \cdots& E[{IPDcount}_{n}] \end{matrix} \right)$

γ is the overdispersion parameter and ***R*** is the correlation matrix for the n *IPDcount* values, with entries:

$Corr\left( I{PDcount}_{t},I{PDcount}_{t+x} \right)=\rho^{x}$

$\mathrm{with} x=0,1,2,\ldots,n-t$ and *ρ* the correlation coefficient

**Section B: Fitting procedure of SARIMA models to whole population and 65 year and older sub-population**

SARIMA models were fitted after stabilising the variance of the time series. A SARIMA model is written as SARIMA(p, d, q)(P, D, Q)_s_, where p,d and q denote the order of autoregressive (AR), differencing and moving average (MA) terms, respectively. The seasonal AR, differencing and MA orders are given by P,D and Q, respectively. The period of seasonality *s*, is 52 weeks since ILI incidence has an annual period. To identify the best-fit SARIMA model, the autocorrelation function (ACF) and partial autocorrelation function (PACF) were inspected, and complemented with the Akaike information criterion (AIC).

Before fitting SARIMA models to the data, the ILI and IPD incidence time series need logarithmic transformation to stabilize the variance. Because incidences may include zero’s, a small constant is added to the data before log-transformation. The transformations are log(incidence+0.25) for the whole population weekly ILI incidence, and log(incidence+1.01) for weekly ILI incidence in the 65+ sub-population. The ACF plot of the ILI incidence in the whole population after log-transformation has positive autocorrelations out to a high number of lags with a seasonal pattern. This suggests the time series is non-stationary, and a 52 week-period seasonal differencing is needed (Fig. S2A). The (p)ACF plots of the differenced series suggest that additional non-seasonal and seasonal parameters are required. (Fig. S3A-B). The pACF plot displays a sharp cut-off and positive auto-correlations for the first 2 lags, indicating non-seasonal AR terms are needed. Besides, in both the ACF and pACF plots, a negative correlation at lag 52 suggests that a seasonal MA term could be added. Different non-seasonal (p,q) and seasonal (P,Q) parameter were applied, yielding in the SARIMA (2,0,0)(0,1,1)_52_ (AIC=397,5) as best fit model based on patterns of the autocorrelations and AIC values (Table S1). The (p)ACF values of the ILI residuals are mostly located within the 95% confidence limits around 0, meaning the best-fit model removed auto-correlations (Fig. S2B-C).

In the older population, the ACF plot of the incidences after log-transformation also shows significant autocorrelations with a seasonal pattern (Fig. S2D), indicating the need for seasonal differencing. The differenced ACF and PACF both show a negative cross-correlation at week 52, suggesting a seasonal MA term might be necessary (Fig.S3C-D). Besides, a non-seasonal AR term could be needed, looking at the sharp cut-off after 1 of 2 lags in the PACF plot. Then, several ARIMA models with different MA and AR terms were fitted (Table S2), resulting in the best-fit SARIMA (1,0,1)(0,1,1)_52_ model (AIC=675,17). The values of the ILI residuals ACF and PACF plots show no auto-correlation, while most values are located within the 95% confidence limits (Fig. S2E-F).

**Figure S2**. ACF and PACF plots for the log-transformed weekly ILI incidence (A) and ILI residuals for the fitted model SARIMA (2,0,0)(0,1,1)_52_ (B+C). Bottom row: 65+ population ACF plot for log-transformed weekly ILI incidence (D) and ILI residuals for the fitted SARIMA (1,0,1)(0,1,1)_52_ model (E+F) The x-axis represents the number of lags. Dashed blue lines indicate 95% confidence interval around 0.


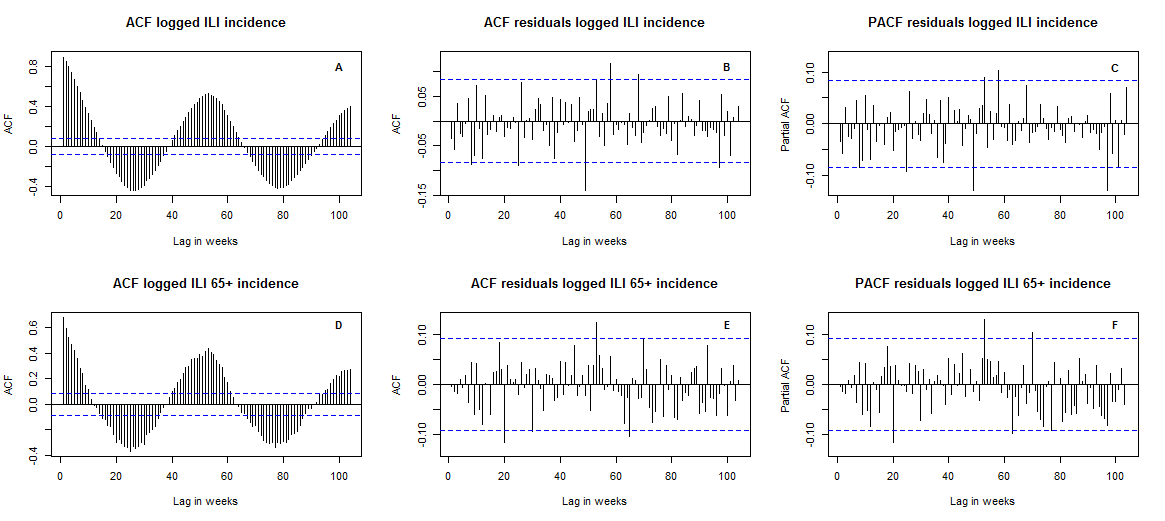


**Figure S3. ACF and PACF plots produced with the seasonal first order differences of log-transformed weekly ILI incidence overall and in the 65+ population.** (A) Autocorrelation function (ACF) and (B) Partial ACF (PACF) plot for overall population. (C) Autocorrelation function (ACF) and (D) Partial ACF (PACF) plot for 65+ population. The x-axis represents the number of lags. Dashed blue lines indicate 95% confidence interval around 0.


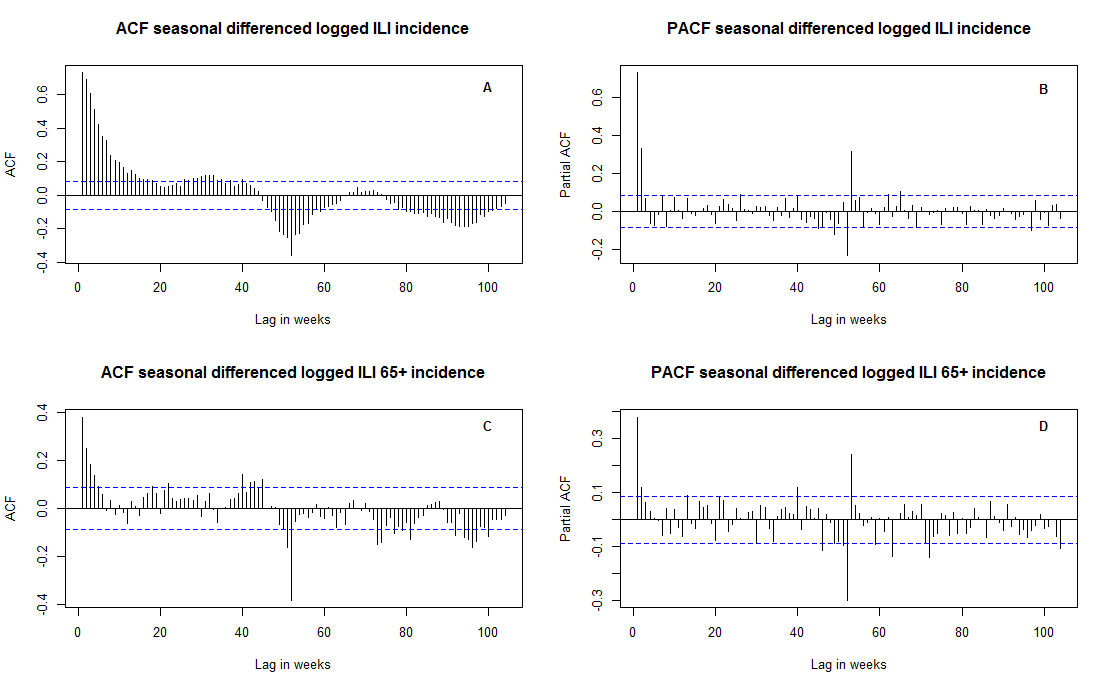


**Table S1. SARIMA models with different parameters on log-transformed weekly ILI incidences.** Non-seasonal parameters (p,d,q) give the order of the autoregressive (AR), differencing and moving average (MA) terms, respectively. The seasonal AR, differencing and MA orders are given by (P,D,Q), respectively. The Akaike information criterion is denoted as AIC. Bold values correspond to the best fit-model.

| Non-seasonal parameters | Seasonal parameters | | AIC | ACF | PACF |
| --- | --- | --- | --- | --- | --- |
| (1,0,0) | (0,1,0)_52_ | 660.12 | | 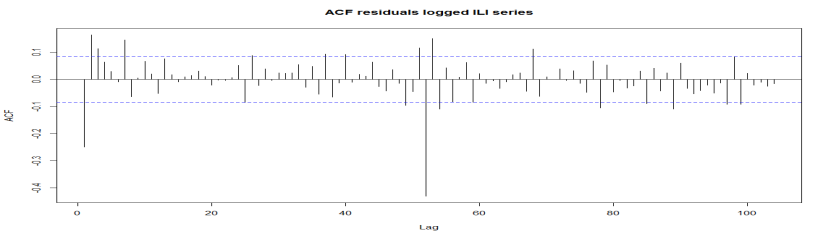 | 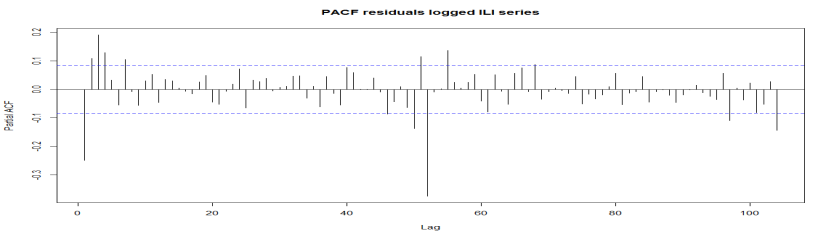 |
| (2,0,0) | (0,1,0)_52_ | 603.41 | | 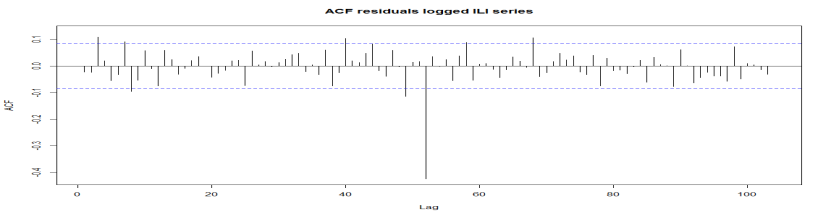 | 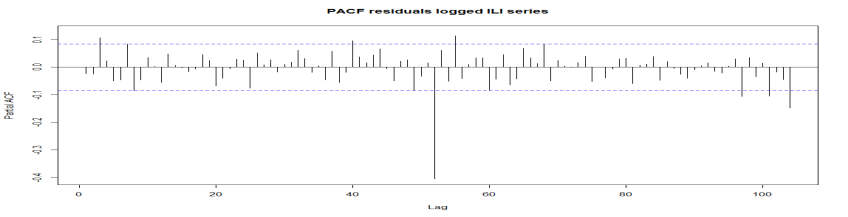 |
| (1,0,1) | (0,1,0)_52_ | 609.63 | | 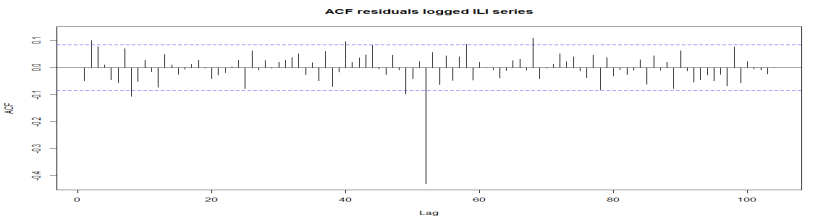 | 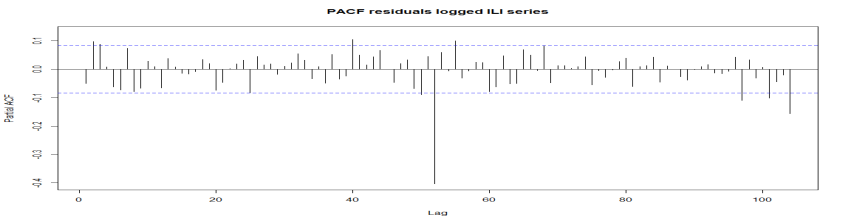 |
| (2,0,0) | **(0,1,1)**_52_ | **397.5** | | 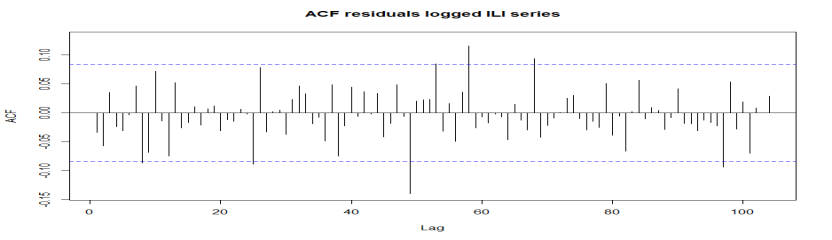 | 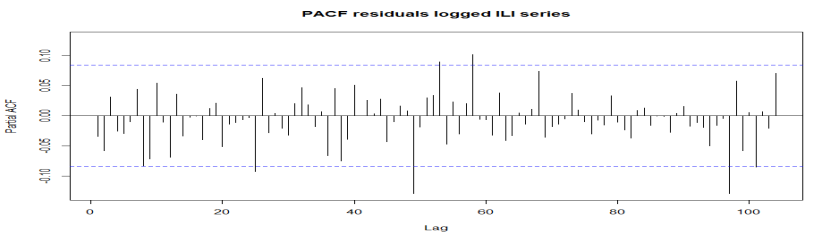 |
| (2,0,0) | (0,1,2)_52_ | 399.5 | | 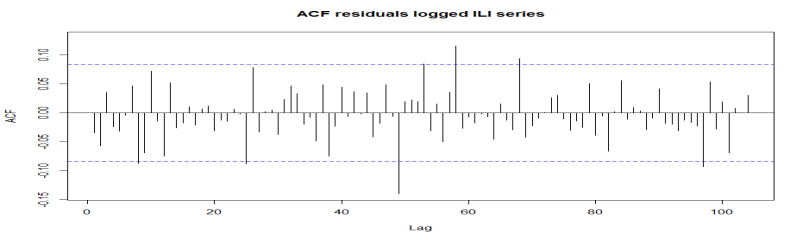 | 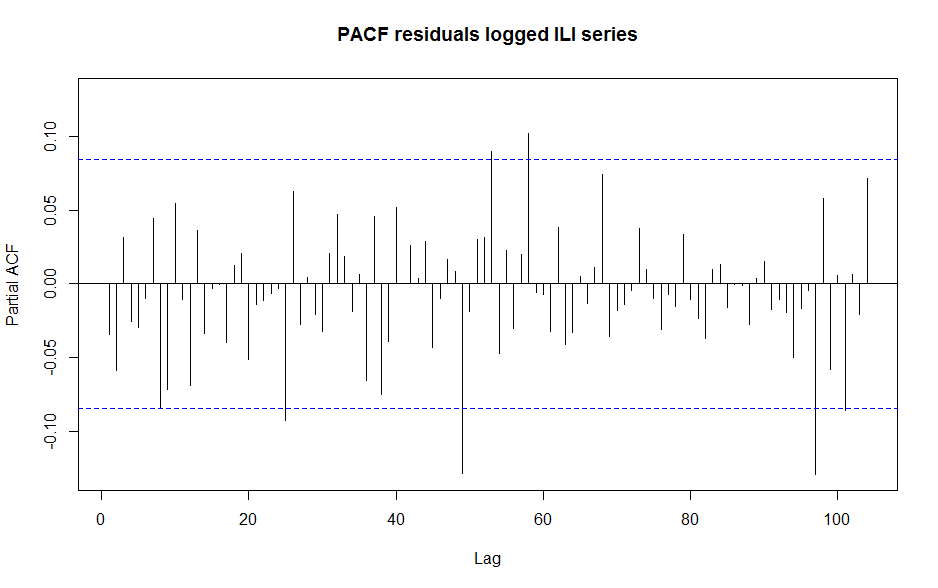 |
| (2,0,1) | (0,1,2)_52_ | 400.15 | | 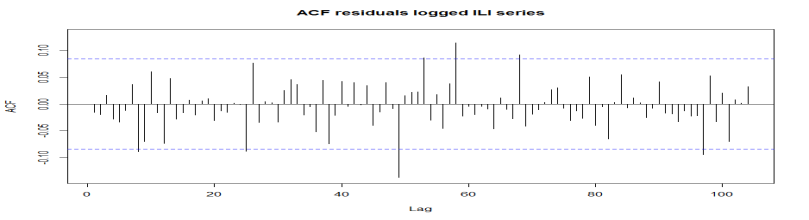 | 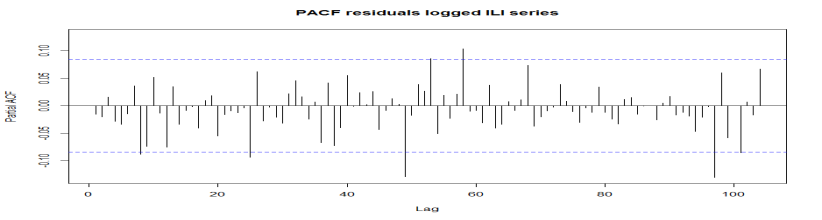 |

**Table S2. SARIMA models with different parameters on logged weekly ILI incidences in 65+ population.** Non-seasonal parameters (p,d,q) denote the order of the autoregressive (AR), differencing and moving average (MA) terms, respectively. The seasonal AR, differencing and MA orders are given by (P,D,Q), respectively. The Akaike information criterion is denoted as AIC. Bold values correspond to the best fit-model.

| Non-seasonal orders | Seasonal orders | AIC | ACF on fit residuals | PACF on fit residuals |
| --- | --- | --- | --- | --- |
| (1,0,0) | (0,1,0)_52_ | 851.17 | 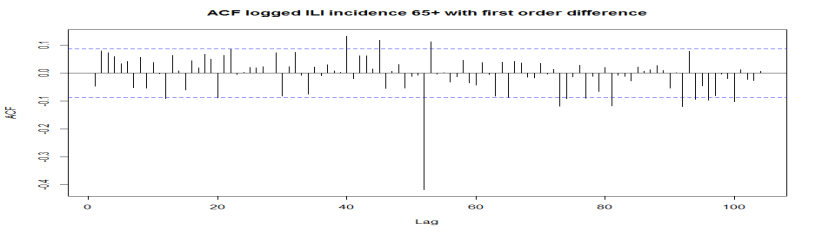 | 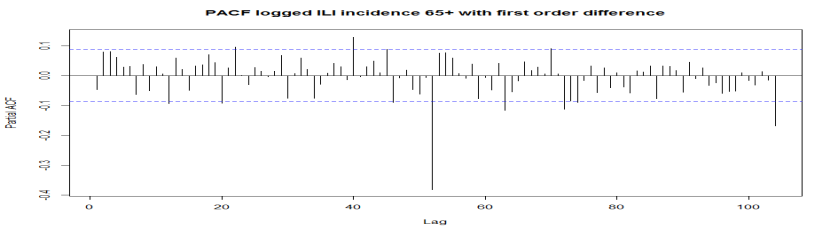 |
| (2,0,0) | (0,1,0)_52_ | 846.38 | 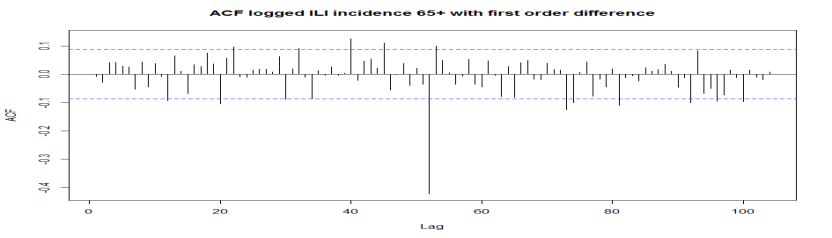 | 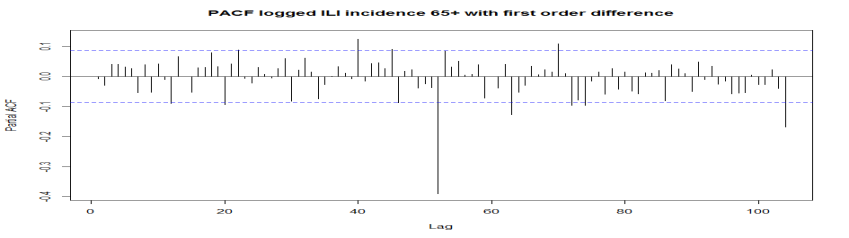 |
| (1,0,0) | (0,1,1)_52_ | 689.68 | 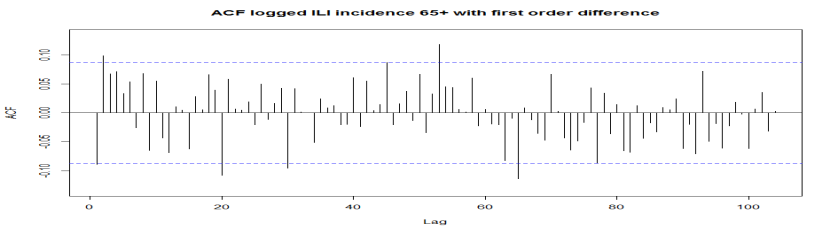 | 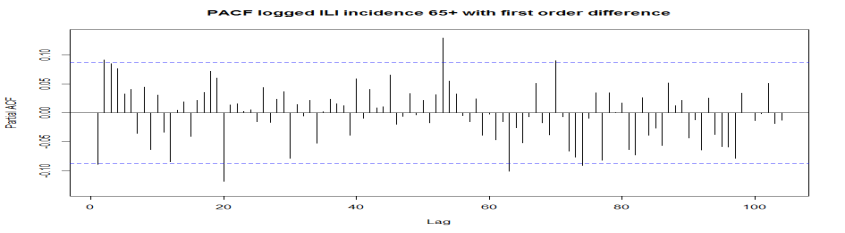 |
| (1,0,1) | **(0,1,1)_52_** | **675.17** | 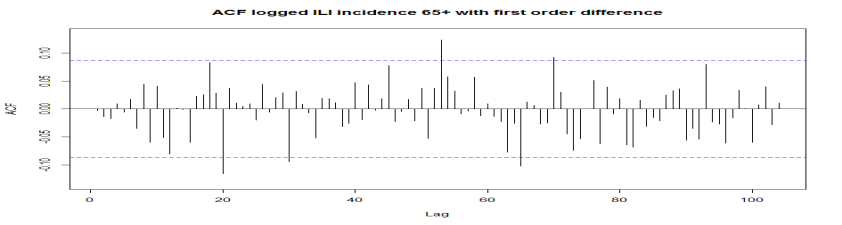 | 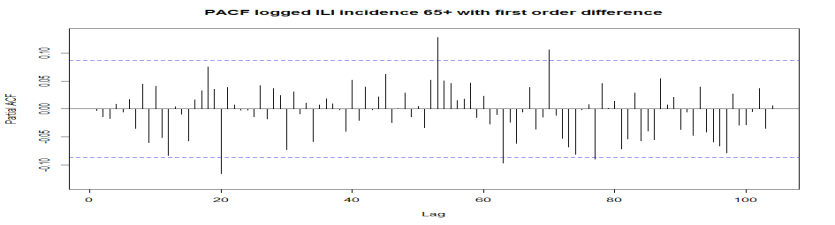 |
| (2,0,0) | (0,1,1)_52_ | 678.06 | 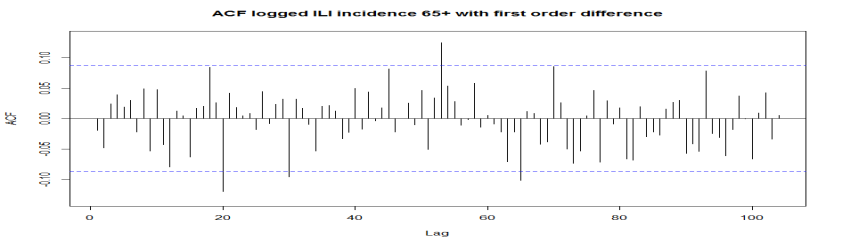 | 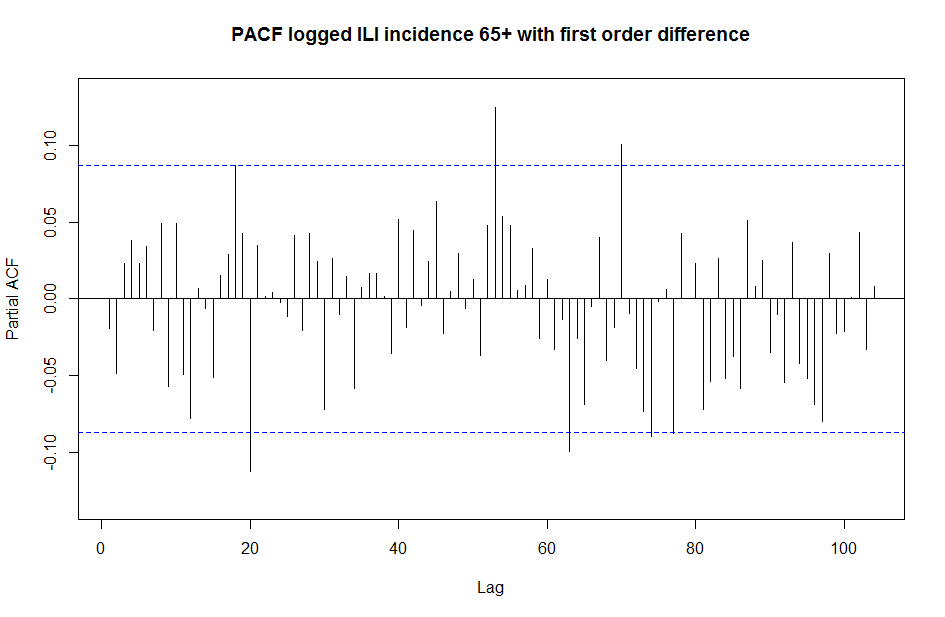 |
| (2,0,0) | (0,1,2)_52_ | 679.98 | 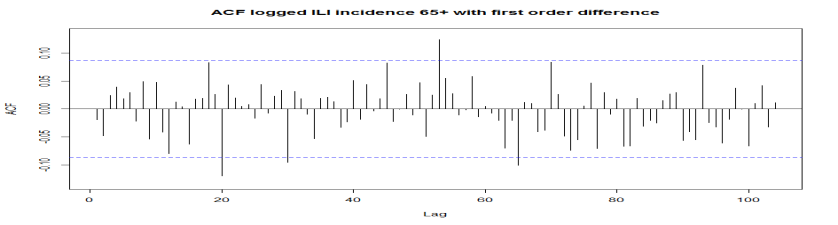 | 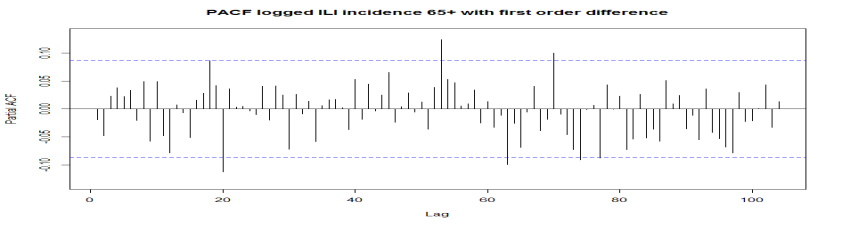 |

**Section C: Prewhitening procedure for cross-correlation**

Prewhitening before carrying out a cross-correlation study is useful when one wants to estimate a possible lag between seasonal and auto-correlated time series (1) (section 11.4: Prewhitening and stochastic regression). The rationale is the following:

1. Fit a time series model (SARIMA) to the independent variable *X_t_*, and store the (white noise) residuals of this in *w_t_*.

2. Filter the dependent Y variable with the best fit model derived in 1. This gives the difference Zt between the observed Y and the “expected Y” under the model for X.

3. The cross-correlation between wt and zt will reveal whether “departures” from the model fitted to the X-variable are related to departures of the Y variable from the expected Y variable corrected for the time series model fitted to X.

In the online tutorial an example is shown (2): when no MA term is included in the best-fit model, the filtering can be done by writing down the equation and solving them (2). These equations become more complex to solve when the best-fit model includes one or more MA terms. As this is the case in our work, we revert to a function in R from the package “forecast”. An example code for this filtering is as follows, for a best-fit ARIMA(1,0,1) to time series X:

Library(forecast)

Fit<-Arima(X, order=c(1,0,1))

Yfiltered<-residuals(Arima(Y, model=fit)

The (sample) cross-correlation is calculated as explained in (1)(section 11.3: Spurious Correlation):

$$r_{k}(X, Y)=\frac{\sum(X_{t}-\bar{X})(Y_{t-k}-\bar{Y})}{\sqrt{{\sum(X_{t}-\bar{X})}^{2}}\sqrt{{\sum(Y_{t}-\bar{Y})}^{2}}}$$

**Supplement D: Plot of residuals**

Fig. S4 Residuals of best-fit SARIMA model to ILI weekly notifications and residuals of filtered (with the best-fit SARIMA) weekly IPD notifications

(A) Whole population, ILI


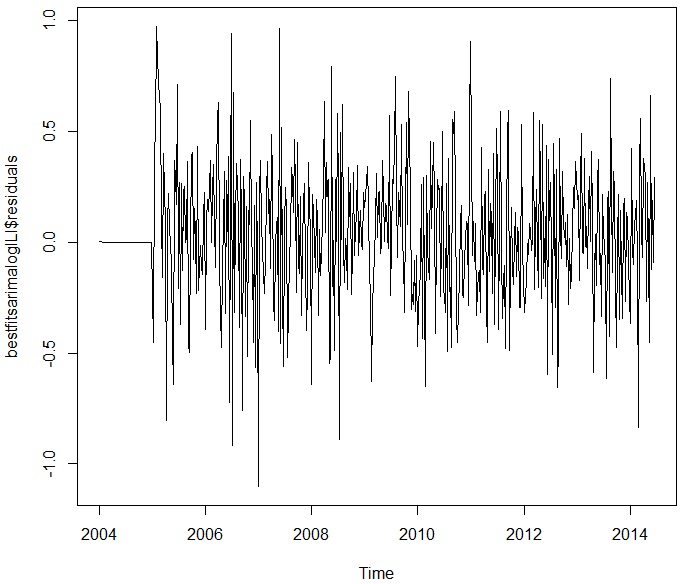


Residuals best-fit SARIMA

(B) Whole population, IPD


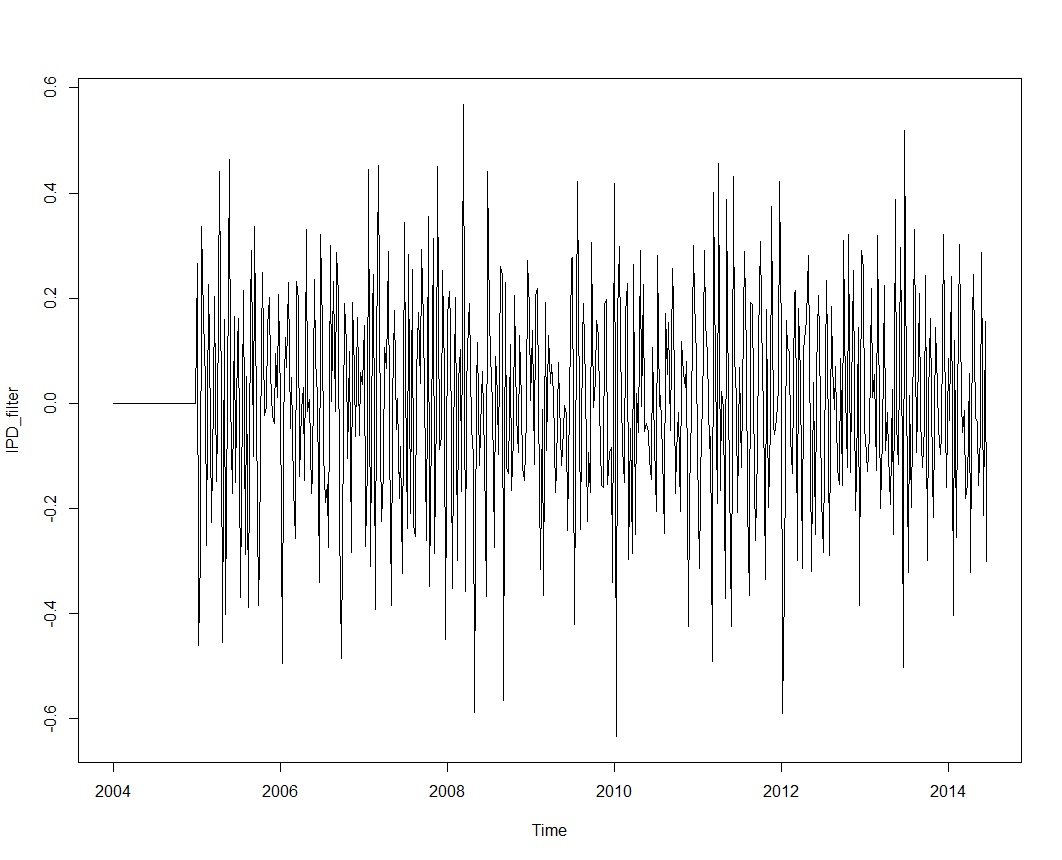


Residuals after filtering


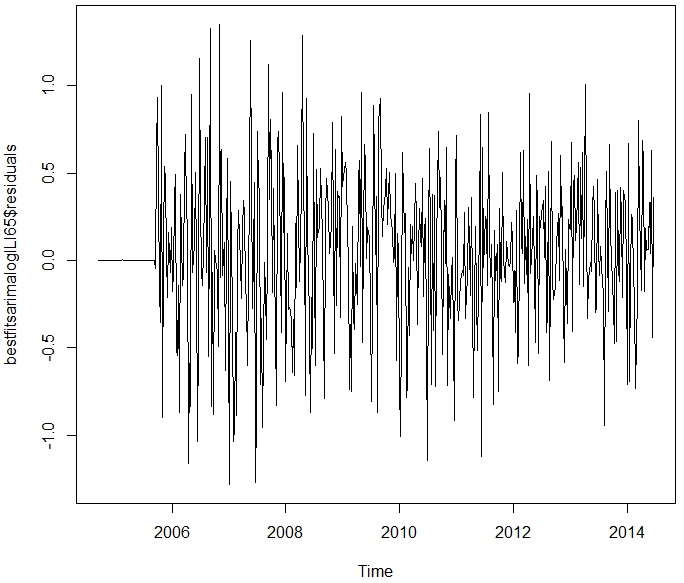


Residuals best-fit SARIMA

(D) Over 65 year-olds, IPD

(C) Over 65-year olds, ILI


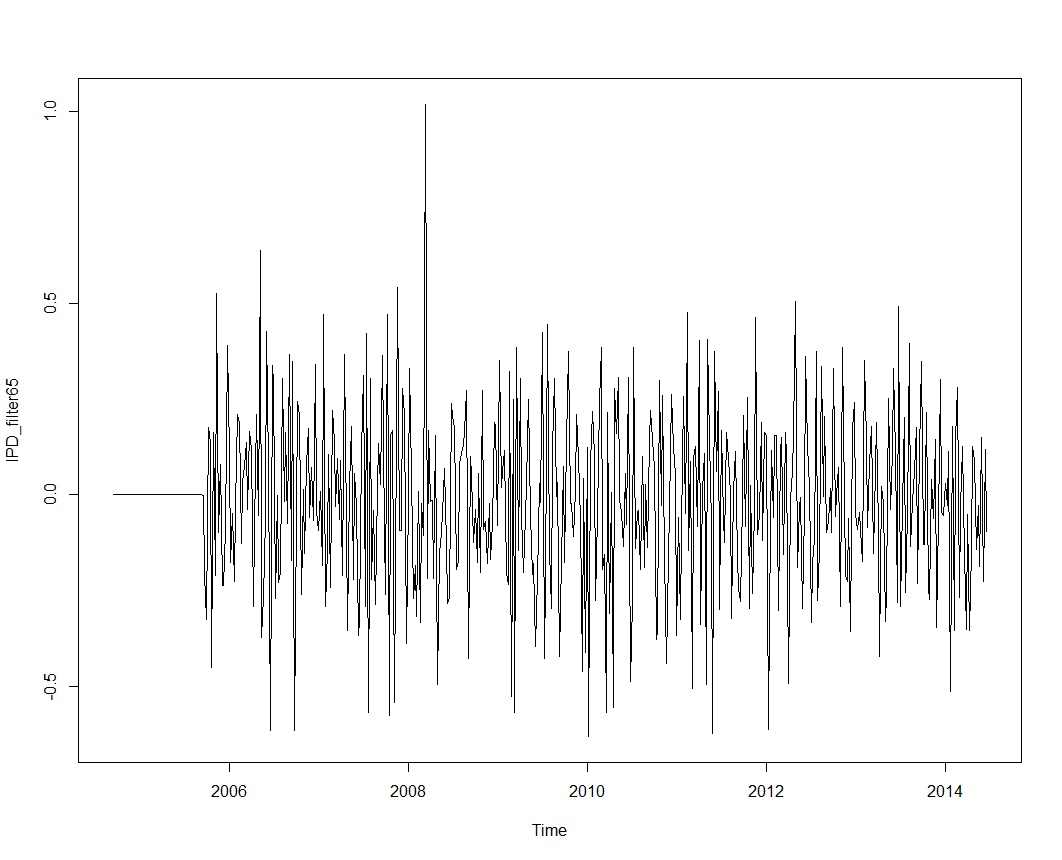


Residuals after filtering

**References**

1. Cryer JD, Chan K. Chapter 11: Time series regression model. Time series analysis with applications in R. 2nd edition ed. New York: Springer-Verlag; 2008.

2. Penn State; Eberly College of Science. Pre-whitening as an Aid to Interpreting the CCF 2016 [updated 2016]. Available from: <https://onlinecourses.science.psu.edu/stat510/node/75>.
